# Supplementary material for: Longitudinal Fluctuations in Protein Concentrations and Higher-Order Structures in the Plasma Proteome of Kidney Failure Patients Subjected to a Kidney Transplant
Source: J Proteome Res. 2024 May 3;23(6):2124–36. doi: 10.1021/acs.jproteome.4c00064 (PMC11165583; doi:10.1021/acs.jproteome.4c00064)
Supplement: Supplementary file 1 — pr4c00064_si_001.pdf [file pr4c00064_si_001.pdf]

Supporting Information

Longitudinal fluctuations in protein concentrations and higher-order structures in the plasma proteome of kidney failure patients subjected to a kidney transplant

Sofia Kalaidopoulou Nteak[1,2], Franziska Völlmy[1,2], Marie V. Lukassen[1,2], Henk van den Toorn[1,2], Maurits A. den Boer[1,2], Albert Bondt[1,2], Sjors P.A. van der Lans[3], Pieter-Jan Haas[3], Arjan D. van Zuilen[4], Suzan H. M. Rooijakkers[3] and Albert J.R. Heck[1,2*]

[1] Biomolecular Mass Spectrometry and Proteomics, Bijvoet Center for Biomolecular Research and Utrecht Institute for Pharmaceutical Sciences, University of Utrecht, Utrecht, 3584 CS, The Netherlands

[2] Netherlands Proteomics Center, Utrecht, 3584 CS, The Netherlands

[3] Department of Medical Microbiology, University Medical Center Utrecht, Utrecht, 3584 CX, The Netherlands

[4] Department of Nephrology and Hypertension, University Medical Center Utrecht, Utrecht University, Utrecht, 3584 CX, The Netherlands

*E-mail: a.j.r.heck@uu.nl

Table of Contents

	Page
**Supplementary Figure 1 | Visualization of variations between the controls and each of the two diseased patients.**	S3
**Supplementary Figure 2 | Plasma concentrations of selected proteins at different timepoints of sampling for each donor.**	S4
**Supplementary Figure 3 | SEC LC-MS elution profile of CRP at distinct timepoint of sampling in P2.**	S5
**Supplementary Figure 4 | SEC LC-MS elution profile of ITIH1, ITIH2 and AMBP.**	S5
**Supplementary Figure 5 | APOA2 and SAA1/SAA2 SEC profiles of P2 at all timepoints and C1 at T1.**	S6
**Supplementary Table 1 | Plasma concentrations of all sampled proteins derived from quantitative proteomics data of the two healthy donors and the two patients at each timepoint of sampling.**	XLSX
**Supplementary Table 2 | Plasma concentrations of all sampled proteins in each of the fractions of the by SEC fractionated sample of patient P2 at all sampled timepoints.**	XLSX

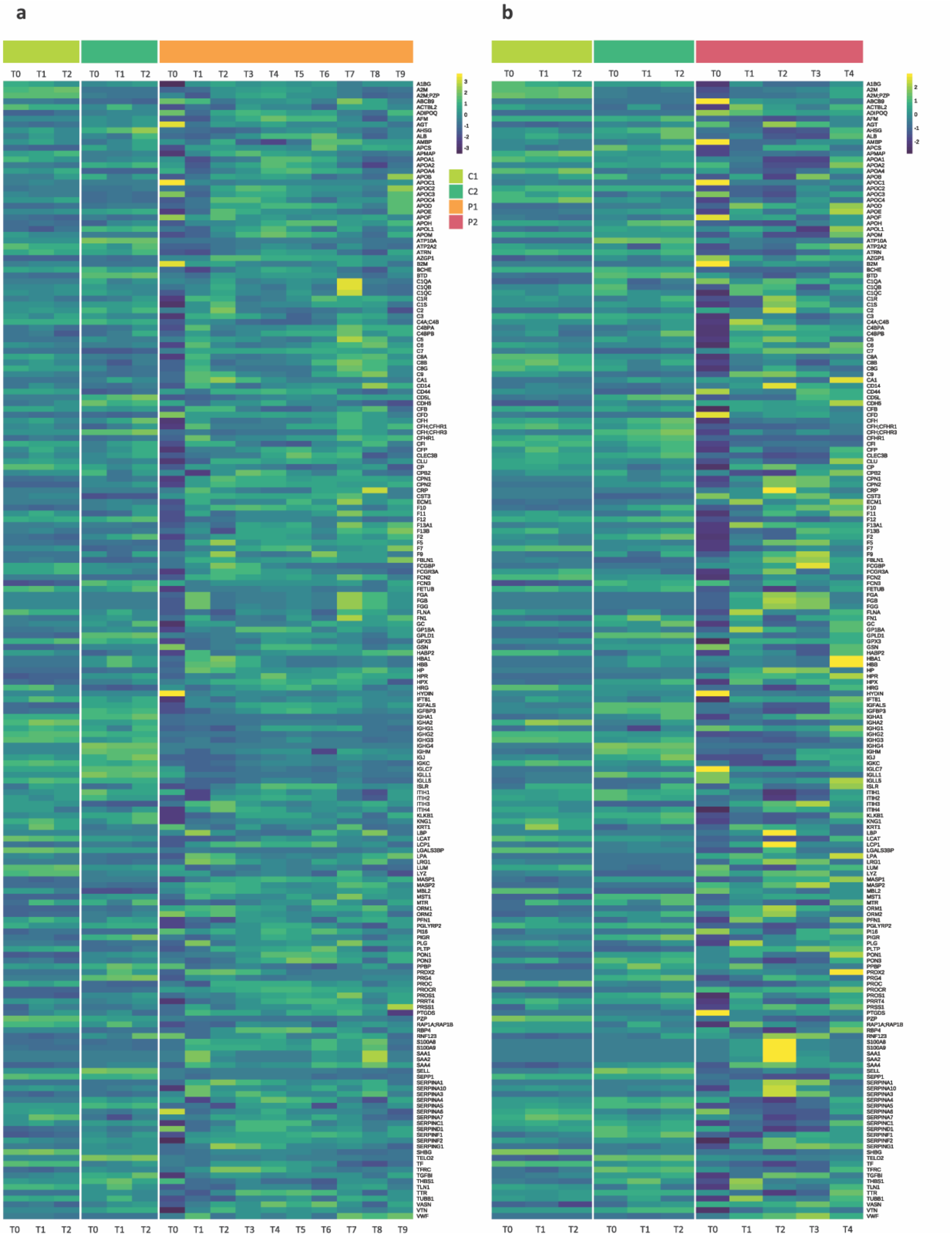

Supplementary Figure 1 | Visualization of variations between the controls and each of the two diseased patients. The heatmaps represent the (z-score) normalized concentrations of the 197 proteins in each timepoint of each sample **a** between the healthy controls and P1 and **b** between the controls and P2. Overall, the healthy profiles are stable over time and differ from the patients. Furthermore, the times of inflammation have distinct profiles in both patients.

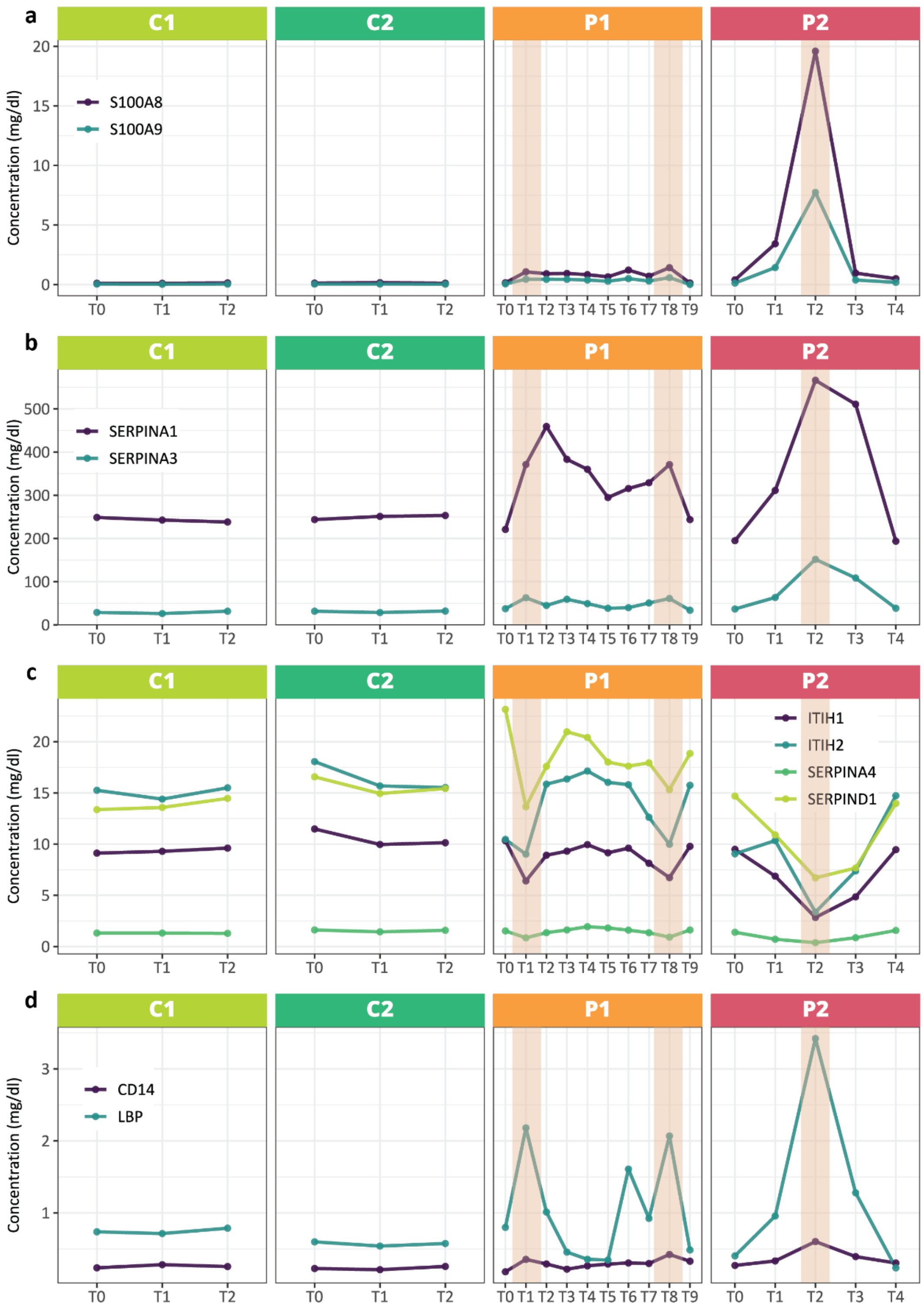

Supplementary Figure 2 | Plasma concentrations of selected proteins at different timepoints of sampling for each donor. The regions highlighted in orange in P1 and P2 indicate the occurrence of bacterial infections. The healthy individuals show relatively stable profiles for all depicted proteins, whereas in the patients during the time of infection there are substantial variations observed.

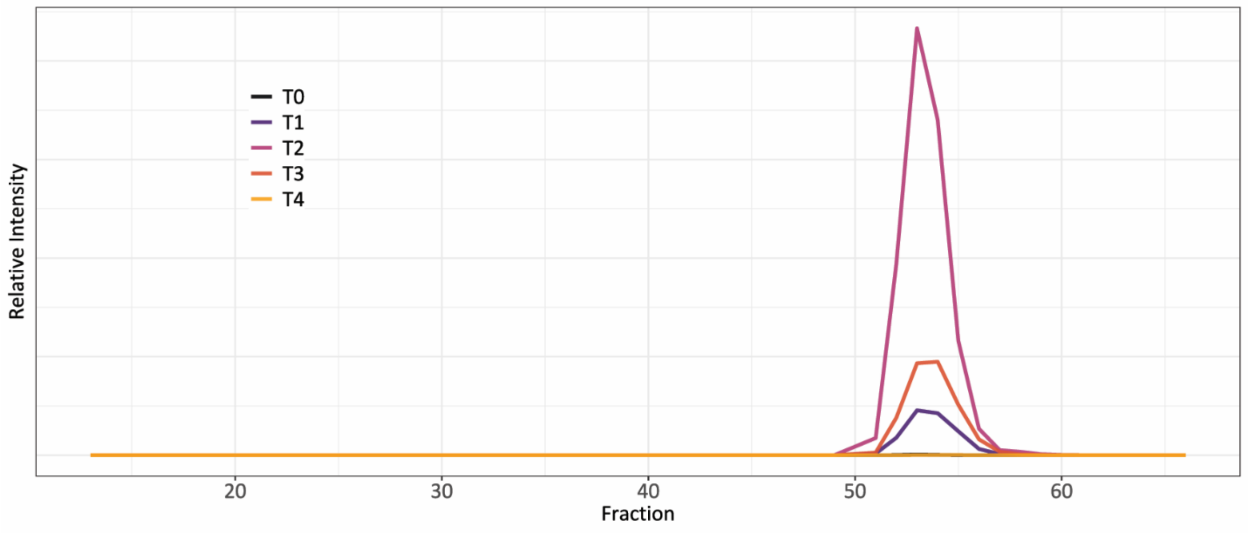

Supplementary Figure 3 | SEC LC-MS elution profile of CRP at distinct timepoint of sampling in P2. CRP elutes around fraction 55, consistent with the mostly pentameric form of CRP known to be present in plasma of ~ 125 kDa.

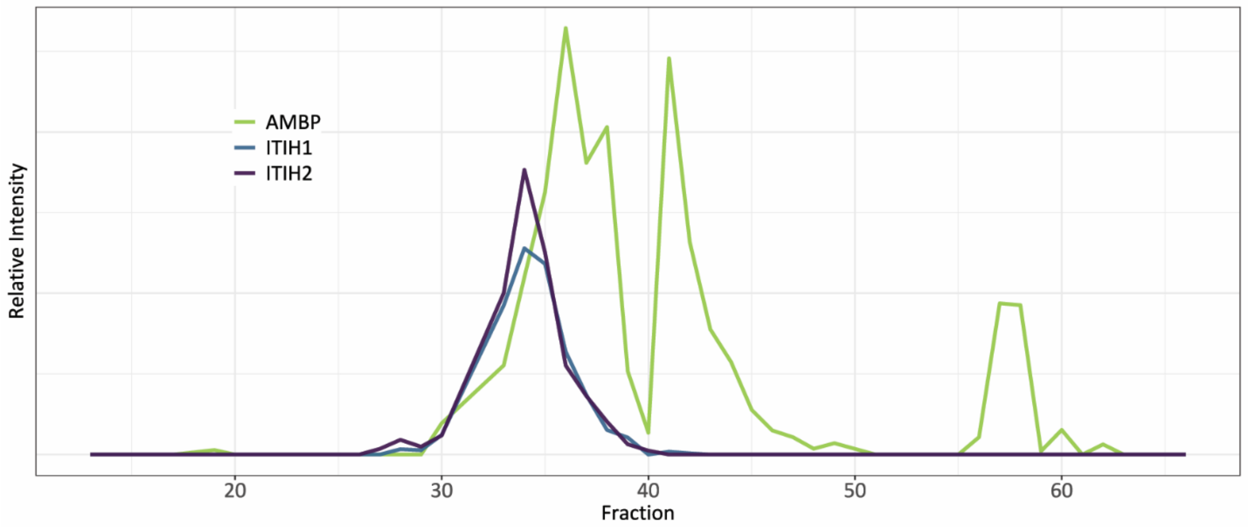

Supplementary Figure 4 | SEC LC-MS elution profile of ITIH1, ITIH2 and AMBP. ITIH1 and ITIH2 perfectly co-elute in fractions between 30 and 40, partially overlapping with the elution profile of AMBP. These three proteins are known to form together a complex in plasma of approximately 225-kDa complex, named IαI, containing the C-terminal fragment of AMBP next to ITIH1 and ITIH2.

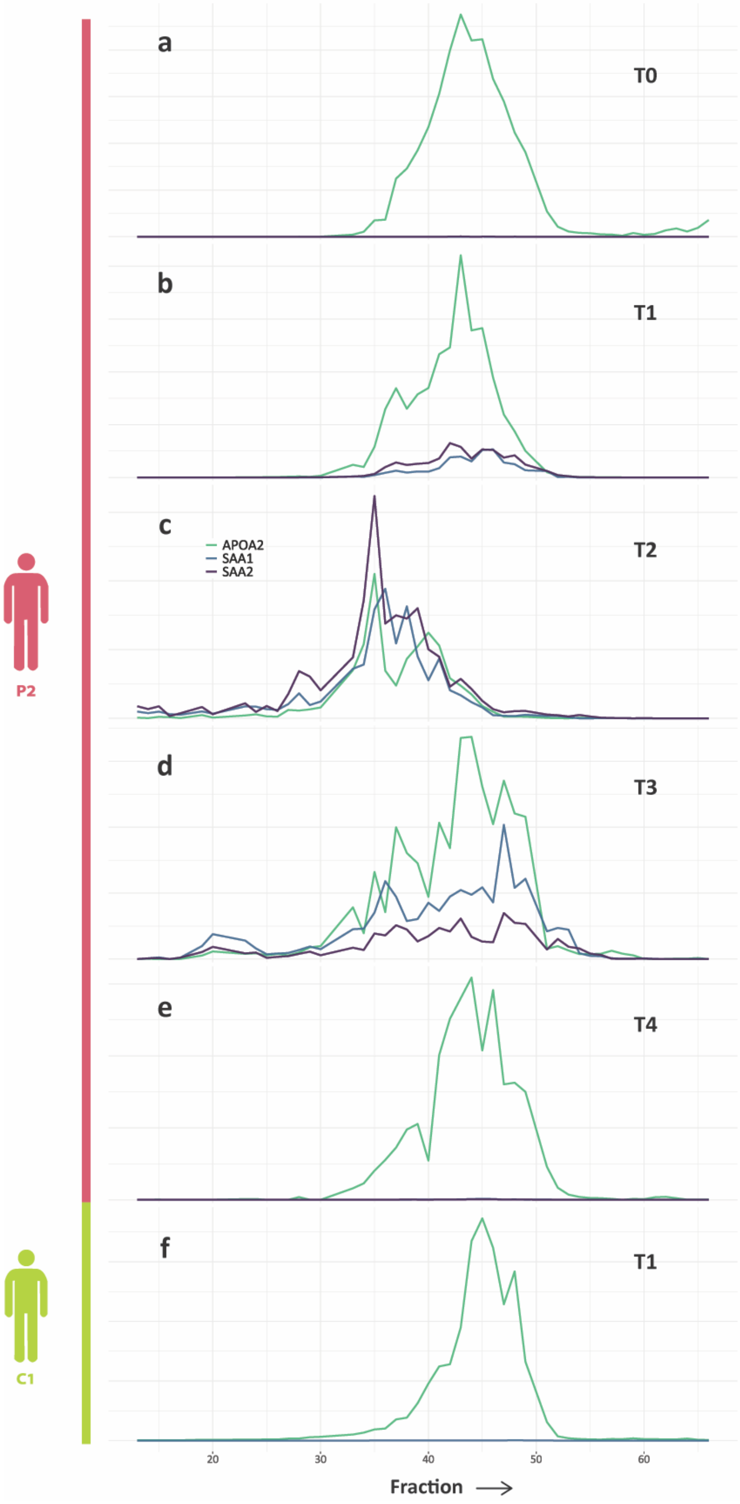

Supplementary Figure 5 | Under APR conditions APOA2, SAA1 and SAA2 co-elute in the SEC LC-MS profiles of samples taken from P2. From top to bottom the SEC LC-MS profiles of APOA2, SAA1 and SAA2 are shown as observed in the samples gathered at the five indicated time points, T0-T4. At the bottom the SEC-LC-MS profile of APOA2 is shown as observed for the control sample C1 at T1. In **a, e** and **f,** the abundances of SAA1 and SAA2 are too low to detect (indicating no APR), but in **b, c** and **d**, at T1, T2 and T3, clear "early" co-elution is observed for APOA2, SAA1 and SAA2, at high molecular weight fractions, being all part of HDL particles.
